# Clustered cardiometabolic risk in pregnancy and dysmenorrhea in offspring: Results from a prospective birth cohort study

**DOI:** 10.1111/eci.70134

**Published:** 2025-10-14

**Authors:** Siyu Zhou, Nikol Shkarpa, Cathy Brouwer, Emmy van den Boogaard, Martijn J. J. Finken, Marcel (Th. B) Twickler, Tanja G. M. Vrijkotte

**Affiliations:** ^1^ Department of Public and Occupational Health, Amsterdam University Medical Centre University of Amsterdam Amsterdam The Netherlands; ^2^ Amsterdam Public Health Research Institute Amsterdam The Netherlands; ^3^ Amsterdam Reproduction and Development Research Institute Amsterdam The Netherlands; ^4^ Department of Endocrinology, Diabetology and Metabolism AZ Monica Antwerp Belgium; ^5^ Faculty of Medicine and Health Sciences University of Antwerp Antwerp Belgium; ^6^ Department of Obstetrics and Gynaecology, Amsterdam University Medical Centre University of Amsterdam Amsterdam The Netherlands; ^7^ Department of Paediatric Endocrinology, Emma Children's Hospital, Amsterdam UMC VU University Amsterdam the Netherlands

**Keywords:** body mass index, clustered cardiometabolic risk, dysmenorrhea, menarcheal age

## Abstract

**Background:**

Dysmenorrhea, a common gynaecological complaint, is often underdiagnosed, particularly in adolescents. The Developmental Origins of Health and Disease hypothesis suggests that maternal cardiometabolic conditions during pregnancy may influence offspring reproductive health. We investigated whether cardiometabolic risk (CCMR) is associated with dysmenorrhea risk in offspring and whether early‐puberty BMI and menarcheal age mediate these associations.

**Methods:**

Data was from the Amsterdam Born Children and their Development cohort. A total of 982 mother‐daughter pairs were included. Maternal CCMR included pre‐pregnancy body mass index (BMI), blood pressure, glucose, triglycerides and Apolipoprotein A1. Dysmenorrhea was defined as menstrual abdominal/back pain requiring analgesics. Inverse probability weighted multivariable logistic regression examined associations between maternal CCMR or its components and dysmenorrhea in offspring. Multiple imputation was used to handle missing data in the sensitivity analysis. Serial multiple mediation analysis tested the mediating role of offspring's BMI and menarcheal age.

**Results:**

Dysmenorrhea was reported in 49.2% of daughters. In the model adjusted for maternal age, socioeconomic status, smoking, alcohol use, anxiety and depressive symptoms, CCMR was not significantly associated with dysmenorrhea (OR: 1.03, 95% CI: .72–1.48). However, higher maternal pre‐pregnancy BMI was associated with increased dysmenorrhea risk in offspring (OR: 1.20, 95% CI: 1.02–1.42). A partial mediation via BMI and menarcheal age was observed (indirect effect: 1.01, 95% CI: 1.00–1.03).

**Conclusion:**

No evidence was found of maternal CCMR and dysmenorrhea in offspring. However, higher maternal pre‐pregnancy BMI increased dysmenorrhea risk, partly mediated by heavier BMI and earlier pubertal timing in offspring. These findings align with the hypothesis of a possible intrauterine origin of menstrual disorders and highlight the importance of early life factors in dysmenorrhea research.

## Graphical abstract

Dysmenorrhea is a leading gynaecological complaint starting from adolescence, yet its developmental origins remain poorly understood. Using data from 982 mother‐daughter pairs in the ABCD cohort, we examined maternal cardiometabolic risk (CCMR) during pregnancy and its association with adolescent dysmenorrhea. While overall CCMR showed no significant link, higher maternal pre‐pregnancy BMI increased dysmenorrhea risk in offspring, partly mediated by higher BMI and earlier menarche. By revealing that dysmenorrhea risk may be influenced before birth, this work highlights the need to consider intergenerational and developmental environments in reproductive research.

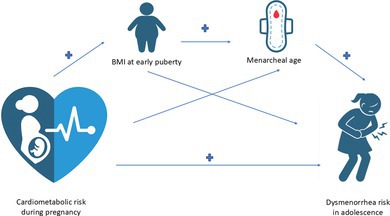

## 1 INTRODUCTION

Dysmenorrhea is characterized by pain occurring before or during menstruation, typically localized in the lower abdomen and often radiating to inner thighs and back. It is one of the most common gynaecological complaints, affecting 45%–95% of females worldwide.^1^, ^2^ Despite its high prevalence, dysmenorrhea is often considered normal, resulting in underdiagnosis, particularly among adolescents.^3^ Untreated dysmenorrhea can impair quality of life, school performance and interpersonal relationships.^4^ Even in the absence of identifiable pathology, dysmenorrhea has been associated with later development of conditions such as endometriosis and infertility.^5^, ^6^

The Developmental Origins of Health and Disease (DOHaD) hypothesis proposes that adverse exposures in early life can interact with development, thereby increasing susceptibility to disease in later life.^7^ Building on this hypothesis, adverse maternal metabolic conditions, including higher pre‐pregnancy BMI and gestational hyperglycaemia, have been associated with cardiometabolic risks in offspring, such as increased adiposity, dyslipidaemia and impaired glucose tolerance.^8^, ^9^, ^10^

These metabolic disturbances can further disrupt endocrine homeostasis, leading to alteration in reproductive maturation such as younger age at menarche.^11^ Evidently, younger menarcheal age, which is more common among girls with higher BMI in early puberty, has been associated with increased risk of dysmenorrhea.^12^, ^13^ From a developmental perspective and driven by coordinated changes in endocrine function, it is putative that suboptimal metabolic status in pregnant women may increase the risk of dysmenorrhea in their daughters. However, the extent to which these prenatal exposures indirectly influence dysmenorrhea risk through BMI in early puberty and menarcheal age, as opposed to being attributable to genetic liability, remains unclear.

Clustered cardiometabolic risk (CCMR), integrating lipid profiles and anthropometric measures, offers a comprehensive assessment of cardiometabolic status.^14^ Recent studies have provided the reliability and validity of CCMR as a reflection of cardiometabolic status in general populations.^15^, ^16^ Although its application in pregnancy is limited, the interrelated nature of metabolic disturbances during pregnancy and their influence on long‐term offspring health supports that CCMR may offer a better representation of maternal metabolic status.

There is a lack of epidemiological or experimental research testing the DOHaD hypothesis of the association between the maternal metabolic environment and the risk of dysmenorrhea in offspring. To address this research gap, we examined whether maternal CCMR and its individual components during pregnancy are associated with dysmenorrhea risk in offspring, and whether these associations are mediated by early‐puberty BMI and menarcheal age.

## 2 MATERIALS AND METHODS

### 2.1 Study design and population

The Amsterdam Born Children and their Development (ABCD) study is a population‐based, longitudinal birth cohort aimed to identify early‐life factors that may influence children's later health outcomes. Women attending their first pregnancy check‐up between January 2003 and March 2004 in Amsterdam were invited to take part in the ABCD study. Of the total 12,373 pregnant women approached, 8261 gave their written consents to participate in the first and follow‐up questionnaire investigations and 4389 gave their written consents to the biomarker study, which included a blood draw.^17^

**FIGURE 1:**
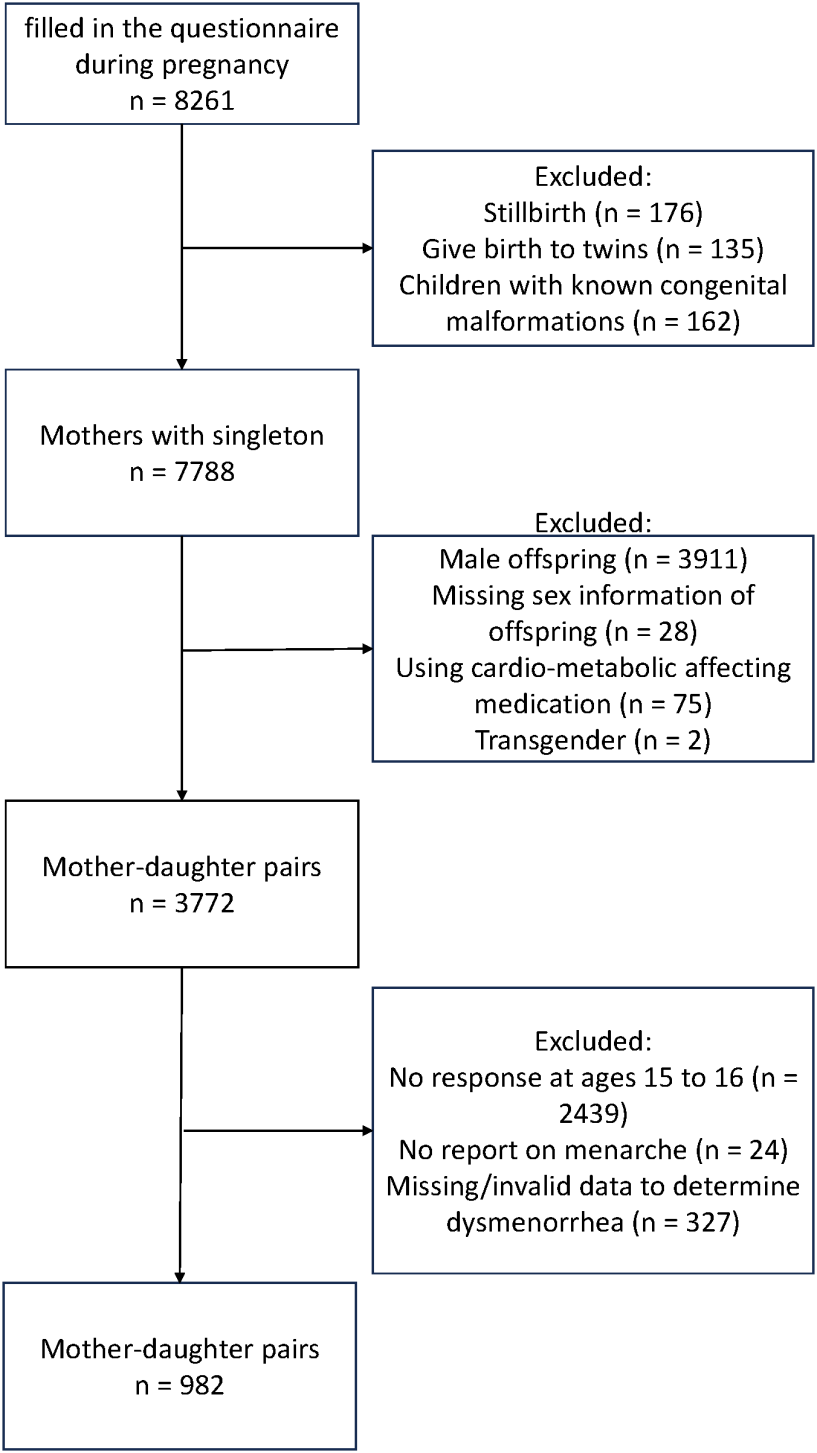
Flowchart of study population selection.

In this analysis, mother‐daughter pairs were included if at least one cardiometabolic profile component was available from the blood draw, along with an available menstrual questionnaire filled out by daughters at age 15–16. We excluded women with multiple births, stillbirths, offspring with known congenital malformations, and those who used medication that may affect their cardiometabolic profile during pregnancy. Additional exclusion criteria were daughters who had not yet experienced menarche by age 15–16 or who identified themselves as transgender. The final study population consisted of 982 mother‐daughter pairs. Figure 1 and Table 2 show the sample sizes for the individual cardiometabolic components.

Approval of the ABCD study was obtained from the Central Committee on Research Involving Human Subjects in The Netherlands (CCMO; number P02.0335 L, 2002), the Medical Research Ethics Committees of the participating hospitals, and the Registration Committee of the Municipality of Amsterdam.

### 2.2 CCMR factors during pregnancy

Determinants of interest in this study were: pre‐pregnancy BMI, systolic blood pressure (SBP), diastolic blood pressure (DBP), random plasma glucose (RPG), random triglycerides (TG), random Apo lipoprotein A1 (ApoA1) and CCMR score.^18^

Pre‐pregnancy BMI was based on the self‐reported pre‐pregnancy height and weight in the pregnancy questionnaire. The SBP and DBP were measured at the first consultation with an obstetric caregiver, and RPG was obtained from medical records at around 12 weeks' gestation. The blood samples were collected at a median of 13 weeks' gestation. Then, serum was obtained from venous blood collected in a 9‐mL Vacuette (Greiner BV, Alphen aan de Rijn, the Netherlands) container. From this sample, levels of TG and ApoA1 were measured. TG was measured with the glycerol‐3‐phosphate oxidase PAP method. ApoA1 was measured with an Abbott Architect CI 8200 analyser (Abbott Laboratories, Limited, Saint‐Laurent, Québec, Canada). The intraassay coefficient of variation was 2.3% for TG, and for ApoA1, it was 1.6%.

The CCMR score in the present study was determined by computing a z‐score for the following components: Pre‐pregnancy BMI, SBP, RGP, TG and ApoA1.^19^ The CCMR score was presented as a continuous variable, representing the average z‐score across the five cardiometabolic risk measurements. Participants who had at least three out of five components were included to calculate CCMR scores. The DBP was excluded from the CCMR score since blood pressure unequally influences the score and thereby, SBP appears to be a better indicator for cardiometabolic risk later in life.^19^ ApoA1 was multiplied by −1, as this component is inversely associated.

### 2.3 Dysmenorrhea in offspring

The primary outcome was dysmenorrhea, assessed by menstrual‐related questionnaires completed at the ages 15 and 16. Dysmenorrhea was defined as abdominal and/or back pain during menstruation requiring the use of medications and/or other hormonal contraceptives for pain management. Adolescent girls who did not meet these criteria were allocated to the group without dysmenorrhea.^12^

### 2.4 Covariates and mediators

Based on previous studies on maternal cardiometabolic health and reproductive health in offspring, the following maternal confounders were considered and obtained from the self‐reported pregnancy questionnaire: age at pregnancy (years), gestational age at blood draw (days), education level (years of education after primary school), ethnicity (Western or non‐Western), smoking during pregnancy (yes or no), alcohol consumption during pregnancy (yes or no), anxiety level (State–Trait Anxiety Inventory (STAI) score)^20^ and depressive symptoms (Centre for Epidemiologic Studies Depression Scale (CES‐D) score).^21^

**FIGURE 2:**
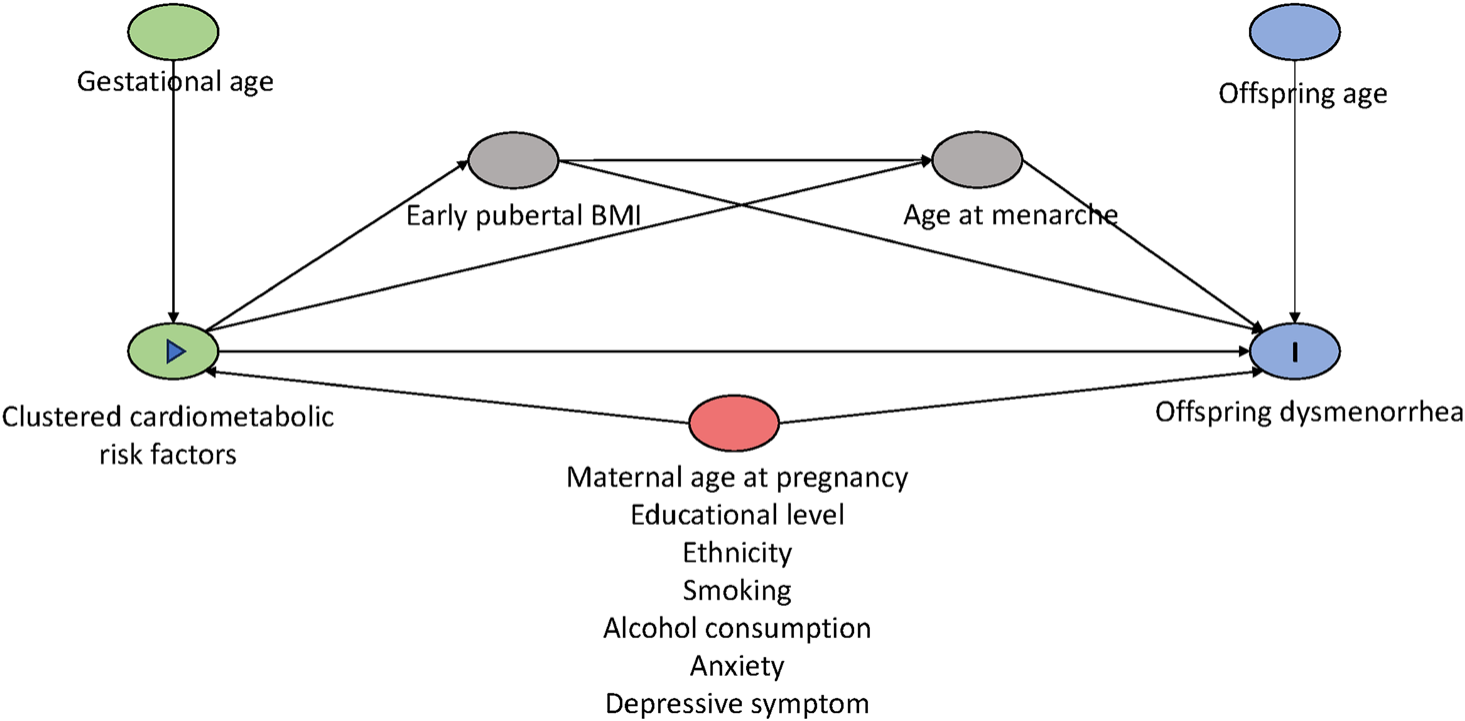
Directed acyclic graph (DAG) illustrating the association between maternal clustered cardiometabolic risk factors and dysmenorrhea in offspring. Green node with array mark represents determinant of interest (maternal clustered cardiometabolic risk factors); Green nodes without mark represents ancestor of the determinant; Red node represents potential confounders (maternal age at pregnancy, educational level, ethnicity, smoking, alcohol consumption, anxiety and depressive symptom); Blue node with letter I mark represents outcome of interest (dysmenorrhea in offspring), Blue node without mark represents ancestor of the outcome, Grey node on the causal path represents potential mediator (BMI in early puberty and age at menarche in offspring).

Daughter's age (years) was calculated by subtracting the date of birth from the date at filling out the menstrual questionnaire. Daughter's BMI in early puberty and self‐reported age at menarche were considered as potential mediators. Daughter's BMI was measured by ABCD health check and youth health care in the Netherlands between 10 and 12 years old; if no measurement was obtained at this age, self‐reported BMI from the questionnaire was used. In Figure 2, a directed acyclic graph (DAG) is shown to depict the potential confounders, covariates and mediators in the association between determinants and outcomes. Detailed descriptions of all non‐blood‐derived and questionnaire‐based variables, including exposures, outcomes and covariates, are presented in Table S1.

### 2.5 Statistical Analysis

First, IBM SPSS 28.0 was used to conduct descriptive statistics (non‐response analysis and characteristics between groups with and without dysmenorrhea) and Pearson correlation analysis. Then multivariable logistic regression analyses were conducted to examine the total effect sizes of CCMR score and its individual components on the risk of developing dysmenorrhea at ages 15–16. To reduce the attrition bias, inverse probability weighting was added, using stabilized weights (calculated as the inverse of the predicted probability of follow‐up participation) derived from baseline maternal characteristics. In the minimally adjusted model (Model 1), gestational age at blood draw and daughter's age at questionnaire completion were adjusted for. Model 2 included additional adjustments for maternal age, ethnicity, educational level, smoking during pregnancy, alcohol consumption during pregnancy, maternal anxiety level and depressive symptoms. All individual CCMR components were standardized to z‐scores. To assess the non‐linearity assumption in models, a quadratic term was added in Model 1. To assess the robustness of our findings, we performed sensitivity analysis with 20 multiple imputed datasets to account for missing baseline data.

Serial mediation analysis was conducted using Model 6 of the SPSS PROCESS macro developed by Hayes.^22^ For this exploratory study, confounders were all included in the serial mediation analysis. A bias‐corrected bootstrap approach with 5000 resamples was used to estimate 95% confidence intervals (95% CI).

## 3 RESULTS

### 3.1 Non‐response analysis

Among the 3772 mother–child pairs followed up, 26% (*n* = 982) were included in the analysis, while the remaining 74% (*n* = 2790) were excluded based on predefined criteria as reported or lost to follow‐up. Non‐response analysis shows that mothers in the non‐responding group were younger at the time of pregnancy (30.2 ± 5.4 vs. 32.2 ± 4.1, *p* < .01), had fewer years of education after primary school (8.1 ± 4.1 vs. 10.5 ± 3.3, *p* < .01), were more likely to be from Non‐Western origin (41.3% vs. 14.9%, *p* < .01), smoked more frequently during pregnancy (10.7% vs. 6.8%, *p* < .01) and consumed alcohol less often during pregnancy (17.9% vs. 29.1%, *p* < .01) compared to responders. There are no statistical differences in CCMR components including pre‐pregnancy BMI, SDP, DBP, RPG, ApoA1 and TG, maternal anxiety score, or depressive symptom score between two groups (Table 1).

**TABLE 1:** Maternal characteristics for the responding and non‐responding group.

	Responding group (*n* = 982)	Non‐responding group (*n* = 2790)
	Mean ± SD/*n* (%)	Mean ± SD/*n* (%)
Maternal age (years)	32.2 ± 4.1	30.2 ± 5.4***
Maternal education (years)	10.5 ± 3.3	8.1 ± 4.1***
Ethnicity		
Western Non‐Western	836 (85.1) 146 (14.9)	1636 (58.7)*** 1146 (41.3)
Smoking during pregnancy		
No Yes	915 (93.2) 67 (6.8)	2491 (89.3)*** 297 (10.7)
Drinking during pregnancy		
No Yes	696 (70.9) 286 (29.1)	2289 (82.1)*** 499 (17.9)
Maternal anxiety (STAI score)	38.3 ± 9.8	38.8 ± 10.5
Maternal depressive symptom (CES‐D score)	12.5 ± 8.2	13.0 ± 8.8
Pre‐Pregnancy BMI (kg/m^2^)	22.8 ± 4.3	22.8 ± 3.5
Blood pressure (mmHg)		
DBP SBP	66.6 ± 8.6 112.7 ± 12.1	66.5 ± 8.7 112.2 ± 12.2
RPG (mmol/L)	4.4 ± 1.0	4.4 ± 1.1
ApoA1 (gram/L)	1.6 ± .2	1.6 ± .2
TG (mmol/L)	1.4 ± .6	1.4 ± .6

*Note*: Difference between two groups was examined using independent *t*‐test for continuous variables and Chi‐square test for categorical variables. Significance level: **p < .05, ***p < .01.

Abbreviations: ApoA1, Apo lipoprotein A1; BMI, body mass index; CES‐D, Centre for Epidemiologic Studies Depression scale; DBP, diastolic blood pressure; RPG, random plasma glucose; SBP, systolic blood pressure; SD, standardized deviation; STAI, the State–Trait Anxiety Inventory; TG, triglycerides.

### 3.2 Descriptive statistics

Dysmenorrhea was present in 483 (49.2%) participants. Maternal and daughter characteristics stratified by dysmenorrhea status are displayed in Table 2. Maternal characteristics were generally similar between the two groups, except for a higher pre‐pregnancy BMI (23.1 ± 3.7 vs. 22.6 ± 3.2 kg/m^2^, *p* < .05) among mothers of daughters with dysmenorrhea. Daughters in both groups had a mean age of 15.9 years. However, daughters with dysmenorrhea had a younger mean menarcheal age (12.5 ± 1.1 vs. 13.0 ± 1.2 years, *p* < .01). Additionally, a higher proportion of daughters with dysmenorrhea reported smoking (8.1% vs. 2.8%, *p* < .01) and alcohol consumption (39.8% vs. 27.7%, *p* < .01) during adolescence, compared to those without dysmenorrhea. Other characteristics did not differ significantly between the groups. The descriptive statistics remained the same after imputation (Table S3).

**TABLE 2:** Maternal and offspring's characteristics in two groups with and without dysmenorrhea.

	Number of samples	Girls with dysmenorrhea (*n* = 483)	Girls without dysmenorrhea (*n* = 499)
		Mean ± SD/*n* (%)/median [IQR]	Mean ± SD/*n* (%)/median [IQR]
*Maternal characteristics*			
Age (years)	982	32.4 ± 4.1	32.0 ± 4.1
Ethnicity			
Western Non‐Western	982	415 (85.9) 68 (14.1)	421 (84.4) 78 (15.6)
Education (years)	981	10.5 ± 3.3	10.5 ± 3.2
Smoking			
No Yes	982	444 (91.9) 39.0 (8.1)	471 (94.4) 28.0 (5.6)
Drinking			
No Yes	982	344 (71.2) 139 (28.8)	352 (70.5) 147 (29.5)
Maternal anxiety (STAI score)	979	36.0 ± 10.2	36.0 ± 9.8
Maternal depressive symptom (CES‐D score)	979	11.1 ± 8.4	11.1 ± 7.8
Pre‐Pregnancy BMI (kg/m^2^)	982	23.1 ± 3.7	22.6 ± 3.2**
Blood pressure (mmHg)			
DBP SBP	869	67.0 (8.2) 112.8 (11.8)	66.1 (8.9) 112.5 (12.4)
Gestational age at blood collection (days)	982	93.0 ± 24.3	90.7 ± 18.5
RPG (mmol/L)	356	4.5 ± 1.0	4.4 ± 1.0
ApoA1 (gram/L)	566	1.6 ± .2	1.6 ± .2
TG (mmol/L)	609	1.3 [.9–1.6]	1.2 [.9–1.6]
CCMR (sum standardized z‐scores)	732	.03 ± .55	−.02 ± .55
*Girls characteristics*			
Age (years)	982	15.9 ± .4	15.9 ± .3
Age at menarche (years)	982	12.5 ± 1.1	13.0 ± 1.2***
BMI at ages 11–12 (kg/m^2^)	827	17.2 ± 2.5	16.9 ± 2.4
Smoking			
No Yes	982	444 (91.9) 39 (8.1)	485 (97.2)*** 14 (2.8)
Drinking			
No Yes	982	291 (60.2) 192 (39.8)	361 (72.3)*** 138 (27.7)

*Note*: Difference between two groups was examined using independent t‐test for continuous variables and Chi‐square test for categorical variables. Significance level: **p < .05, ***p < .01.

Abbreviations: ApoA1, Apo lipoprotein A1; BMI, body mass index; CCMR, clustered cardiometabolic risk score; CES‐D, Centre for Epidemiologic Studies Depression scale; DBP, diastolic blood pressure; IQR, interquartile range; RPG, random plasma glucose; SBP, systolic blood pressure; SD, standardized deviation; STAI, the State–Trait Anxiety Inventory; TG, triglycerides.

### 3.3 Correlation among the determinants

Table 3 presents the correlations among the determinants. The correlations between individual CCMR components are negligible or not observed, except for a correlation between DBP and SBP (*r* = .55).

**TABLE 3:** Correlations matrix of individual cardiometabolic risk components and clustered scores.

	Pre‐pregnancy BMI	SBP	DBP	RPG	ApoA1	TG	CCMR
Pre‐pregnancy BMI	1						
SBP	.14	1					
DBP	.19	.55	1				
RPG	.19	−.05	−.01	1			
ApoA1	−.10	.11	.09	.04	1		
TG	.10	.13	.10	.18	.21	1	
CCMR	.66	.55	.35	.52	−.39	.51	1

Abbreviations: ApoA1, Apo lipoprotein A1; BMI, body mass index; CCMR, clustered cardiometabolic risk; DBP, diastolic blood pressure; RPG, random plasma glucose; SBP, systolic blood pressure; TG, triglycerides.

### 3.4 Effect sizes of CCMR and its components on the risk of dysmenorrhea

Results from the multivariate logistic regression analyses are presented in Table 4. Maternal CCMR showed no association with the risk of dysmenorrhea in both models: model 1 (OR: 1.02, 95% CI (.73, 1.44)), adjusted model 2 (OR: 1.03, 95% CI (.72, 1.48)). In both models, pre‐pregnancy BMI (z‐score) was found to be positively associated with the risk of dysmenorrhea in offspring. Adolescent girls were 20% more likely to develop dysmenorrhea if their mothers had one unit increase in pre‐pregnancy BMI (z‐score) (OR: 1.20, 95% CI (1.02, 1.42); *p* = .03) (Table 4). Results of sensitivity analyses based on 20 imputed datasets are presented in Table S4. After imputation, the associations between CCMR and its components were similar to the results of complete case analysis.

**TABLE 4:** Results of the multivariable logistic regression analysis of the association between individual and clustered maternal cardiometabolic risk z‐scores (continuous) and the risk of dysmenorrhea in offspring aged 15 and 16.

	Model 1			Model 2		
	OR	95% CI	*p* Value	OR	95% CI	*p* Value
CCMR (sum z‐score)	1.02	.73–1.44	.90	1.03	.72–1.48	.86
Pre‐pregnancy BMI (z‐score)	1.14	.98–1.32	.08	1.20	1.02–1.42	.03**
DBP (z‐score)	1.12	.94–1.33	.21	1.10	.92–1.31	.32
SBP (z‐score)	.97	.80–1.16	.72	.94	.79–1.13	.94
RPG (z‐score)	.99	.76–1.29	.95	.96	.73–1.27	.78
ApoA1 (z‐score)	.89	.71–1.11	.29	.94	.75–1.19	.61
lnTG (z‐score)	.95	.76–1.19	.66	.94	.75–1.19	.94

*Note*: Model 1: adjusted for gestational age of mothers at determinants measurements and age of girls at outcome measurement. Model 2: adjusted for covariates in model 1 and maternal age at pregnancy, ethnicity, smoking/drinking behaviour, educational level, anxiety and depressive symptom scores. Significance level: **p < .05.

Abbreviations: ApoA1, Apo lipoprotein A1; CCMR, clustered cardiometabolic risk; CI, confidence interval; DBP, diastolic blood pressure; lnTG, natural log triglycerides; OR, odds ratio; Pre‐pregnancy BMI, pre‐pregnancy body mass index; RPG, random plasma glucose; SBP, systolic blood pressure.

### 3.5 Testing serial mediation analysis

**FIGURE 3:**
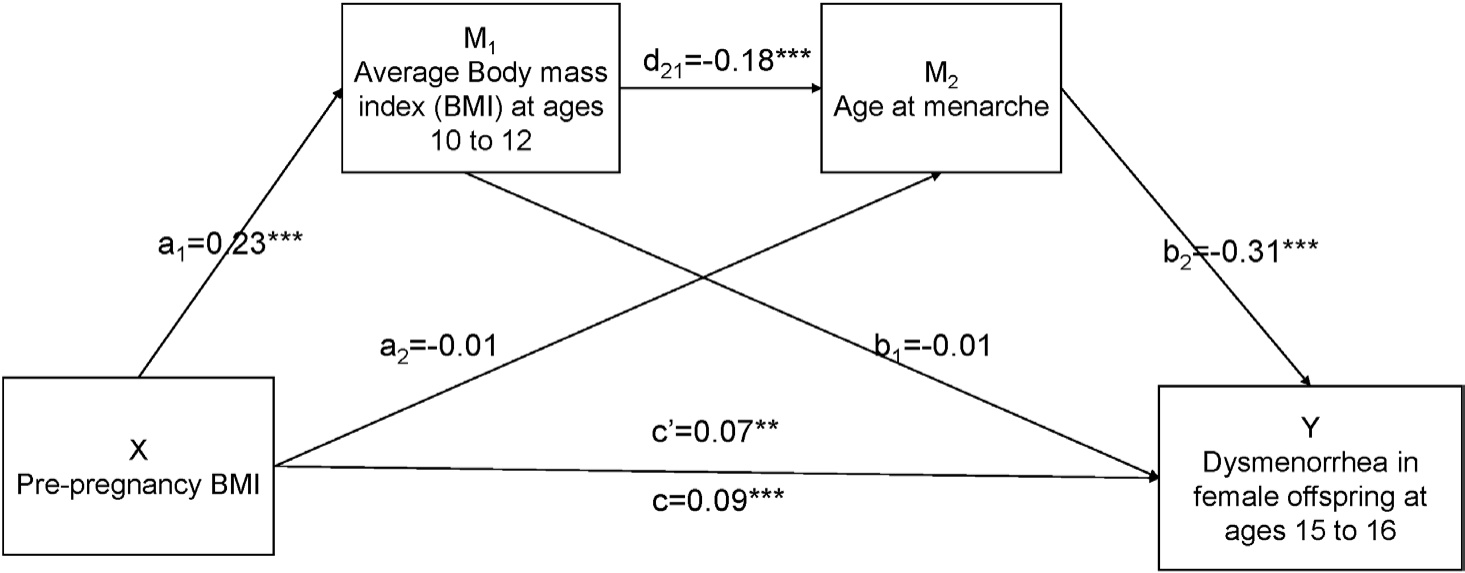
Serial mediation model of the association between maternal pre‐pregnancy body index mass (BMI) and the risk of dysmenorrhea in offspring. The mediation analysis was adjusted for maternal age at pregnancy, educational level, ethnicity, gestational age at measurement, ethnicity, smoking during pregnancy, drinking during pregnancy and daughter's age at questionnaire completion, effects were reported as standardize values. a_1_: Coefficient that describes the change in offspring body mass index (BMI) (kg/m^2^) in early puberty caused by per one unit increase in maternal pre‐pregnancy BMI (kg/m^2^). a_2_: Coefficient that describes the change in offspring age at menarche (years) caused by per one unit increase in maternal pre‐pregnancy BMI (kg/m^2^). d_21_: Coefficient that describes the change in offspring age at menarche (years) caused by per one unit increase in their BMI (kg/m^2^). b_1_: Coefficient that describes the change in offspring risk of dysmenorrhea caused by per one unit increase in their BMI (kg/m^2^). b_2_: Coefficient that describes the change in offspring risk of dysmenorrhea caused by per one unit increase in their age at menarche (years). c': Total indirect effect of maternal pre‐pregnancy BMI on the risk of dysmenorrhea in offspring through all three mediation pathways. c': Direct effect of maternal pre‐pregnancy BMI on the risk of dysmenorrhea in offspring. Significance level: **p < .05, ***p < .01.

In the multivariate logistic regression, only maternal pre‐pregnancy BMI was identified to be associated with the risk of dysmenorrhea with a significant effect size. The serial mediation model between maternal pre‐pregnancy BMI and the risk of dysmenorrhea in offspring is shown in Figure 3. The total effect of pre‐pregnancy BMI on the risk of dysmenorrhea was significant (*β* = .09, *p* < .01). After controlling for confounders, the indirect path indicated that higher maternal pre‐pregnancy BMI was associated with a higher BMI in early puberty (*β* = .23, *p* < .01) in offspring. Next, BMI in early puberty negatively predicted menarcheal age (*β* = −.18, *p* < .01). In turn, younger menarcheal age was associated with a higher risk of dysmenorrhea in offspring (*β* = −.31, *p* < .01). The direct effect of maternal pre‐pregnancy BMI on the risk of dysmenorrhea in offspring remained significant (*β* = .07, *p* < .05) after considering the mediating function, suggesting partial mediation.

### 3.6 Bootstrap test for mediating effect

The mediating effects for each path are presented in Table 5. Among all pathways from maternal pre‐pregnancy BMI to dysmenorrhea in offspring, the total effect and direct effect were significant before and after considering mediation (effect sizes: 1.09, 95% CI (1.03, 1.16) and 1.07, 95% CI (1.00, 1.15), respectively). Among the indirect pathways, the serial mediation pathway showed a small but significant mediation through both BMI in early puberty and menarcheal age (effect size: 1.01, 95% CI (1.00, 1.03)). In contrast, there was no evidence of indirect effects through BMI in early puberty alone and menarcheal age alone (effect sizes: 1.00, 95% CI (−1.03, 1.03) and 1.00, 95% CI (−1.01, 1.02), respectively).

**TABLE 5:** The effects of pathways in the serial multiple mediation analysis of the association between maternal pre‐pregnancy body mass index (BMI) and the risk of dysmenorrhea in offspring.

	Effect size	Bootstrapping SE	Bootstrapping 95% CI
Total effects	1.09	1.03	(1.03, 1.16)
Direct effects (X → Y)	1.07	1.04	(1.00, 1.15)
Indirect (X → M_1_ → Y)	1.00	1.02	(−1.03, 1.03)
Indirect (X → M_2_ → Y)	1.00	1.01	(−1.01, 1.02)
Indirect (X → M_1_ → M_2_ → Y)	1.01	1.01	(1.00, 1.03)

Abbreviations: CI, confidence interval; M_1_, first mediator (early pubertal BMI in offspring); M_2_, second mediator (age at menarche in offspring); SE, standard error; X, independent variable (maternal pre‐pregnancy body mass index (BMI)); Y, outcome variable (dysmenorrhea in offspring).

## 4 DISCUSSION

Our findings suggest that maternal CCMR is not associated with the risk of dysmenorrhea in their offspring aged 15–16, however, among individual CCMR components, maternal pre‐pregnancy BMI is associated with the risk of dysmenorrhea. Specifically, higher maternal pre‐pregnancy BMI (z‐score) was associated with increased risk of dysmenorrhea. Notably, this association was partially mediated in sequence by offspring's BMI in early puberty and menarcheal age, even after controlling for potential confounders. Our observations align with the DOHaD hypothesis that foetal exposure to adverse metabolic conditions has a long‐term influence on offspring reproductive outcome.

To our knowledge, this is the first intergenerational study exploring the association between maternal CCMR and the risk of dysmenorrhea in offspring, with a potential mediating pathway via BMI in early puberty and menarcheal age. Previous studies have consistently linked maternal metabolic disturbances, particularly increased pre‐pregnancy BMI and gestational hyperglycaemia, to offspring obesity and earlier pubertal onset.^23^, ^24^ These results align with our current observations. The biological mechanisms underlying these associations have been extensively explored: there has been growing interest in the role of meta‐inflammation (or chronic, low‐grade metabolically induced inflammation) in developmental programming in foetuses exposed to maternal adverse CCMR.^24^ Unlike acute inflammatory responses, meta‐inflammation is induced by excessive consumption of nutrients and is perpetuated by metabolically active cells such as adipocytes.^25^ Various aspects of maternal and offspring health have been previously discussed.^26^, ^27^, ^28^ Evidence has shown that meta‐inflammation in utero may trigger inflammatory responses in the foetus. For instance, a study in sheep showed that maternal obesity upregulated the TLR4/NF‐κB signalling, promoting adipogenesis and insulin resistance in foetal skeletal muscle during late gestation.^29^ Similarly, a rodent study found that even if foetal body weights were similar, foetuses of high‐fat diet feeding mice exhibited elevated levels of plasma glucose and insulin accompanied by increased expression of inflammatory factors (e.g., CD68, CD192, TNF‐α mRNA).^30^ In humans, newborns of overweight women with gestational diabetes were more likely to display a systemic inflammation manifested by increased levels of IL‐6, IL‐8, ICAM3, TNFR1 and VEGFR2, creating a milieu that predisposes to obesity.^31^ This evidence suggests that maternal CCMR‐induced inflammation can partly explain the intergenerational transmission of metabolic disturbances, along with other contributing factors such as genetic predispositions related to obesity susceptibility and shared familial environments. Moreover, while the mechanism has not yet been elucidated, studies suggest that the association between maternal CCMR and menarcheal age in offspring could be explained by alterations in the settings of the hypothalamus‐pituitary‐gonadal axis and accelerated childhood adiposity gain.^32^, ^33^, ^34^

While previous research has largely focused on metabolic outcomes and pubertal timing, the long‐term reproductive consequences of maternal CCMR, particularly regarding menstrual health, remain underexplored. Our study contributes to the limited body of literature by providing human evidence supporting intrauterine origins of dysmenorrhea. One proposed mechanism supporting this association involves impaired mitochondrial steroidogenesis.^35^ This dysfunction may alter sex steroid hormone levels in offspring, thereby affecting different aspects of reproductive function involving menstrual health. While it is still hypothetical, more experimental studies are certainly warranted to unravel the underlying mechanisms between maternal CCMR and the risk of dysmenorrhea in offspring.

Associations between higher BMI in early puberty, younger menarcheal age sequence and dysmenorrhea risk have been found in some studies^(^ ^11^, ^36^). However, inconsistent results in other studies also reflect the complexity of this pathway.^32^, ^33^, ^34^ In our study, the indirect pathway only explained around 11% of the association. Therefore, future population‐based studies exploring potential (genetic and environmental) pathways may further uncover the underlying mechanism between maternal CCMR and the risk of dysmenorrhea in offspring.

The greatest strength of this study lies in its prospective design, which enabled testing of temporal relationships, thereby enhancing the ability to infer potential causality. In the present study, data were reported or collected at the time of the event from pregnancy to offspring adolescence. The lag time between the event and reporting was short, which may minimize recall bias, thereby increasing data accuracy. However, several limitations should be acknowledged. First, the definition of dysmenorrhea, menstrual pain in the lower abdomen and/or back requiring medication, may have captured predominantly moderate to severe cases of dysmenorrhea. Our observed prevalence of 49.2% is consistent with the lower range of global estimates (ranging from 45% to 95%).^1^ For future studies, it is suggested to use a numerical rating scale to distinguish between mild and severe dysmenorrhea based on the necessity of medical intervention. Second, at cohort initiation, certain reproductive history factors (such as menarcheal age and dysmenorrhea history) and paternal characteristics (including BMI) were not collected. Such factors represent important unmeasured confounders that may directly and/or indirectly contribute to the risk of dysmenorrhea. Although it is likely that genetic heritability and shared environmental factors could explain part of observed associations, we were only able to adjust for factors such as ethnicity, socioeconomic status, lifestyle factors and psychological factors, which have previously been reported to influence dysmenorrhea risk.^23^ We acknowledge this as a limitation and highlight the need for future studies to incorporate paternal BMI and broader reproductive history in order to provide a more comprehensive assessment of intergenerational influences. In addition, selection bias toward a healthier population may have occurred at cohort initiation. As reported in our previous study,^37^ although lipid profiles in our samples were comparable to those of other cohorts in the first trimester, the lower prevalence of gestational diabetes and hypertensive disorders indicated a relatively healthy population. This underrepresentation of higher‐risk individuals may have underestimated the strength of the observed associations. Finally, in our study, there were several sources of measurement error. Blood was analyzed from random samples and pre‐pregnancy BMI was based on self‐reported height and weight. However, data show that self‐reported pre‐pregnancy weight is on average only 1.3 kg less than measured weight, with underreporting being higher among women with a higher BMI category, late prenatal care entry and pregnancy outcomes other than live/stillbirth.^38^ As the women in our sample were leaner, entered prenatal care at an early stage and gave birth to live children, we believe that the impact of self‐report is unlikely to materially influence our observations. Likewise, imprecision in levels of TG and glucose is unlikely to explain our observations.

To conclude, we demonstrate for the first time that maternal CCMR has a non‐significant tendency to predict a higher risk of dysmenorrhea in offspring, with evidence of a significant association between higher pre‐pregnancy BMI and dysmenorrhea risk. This intergenerational effect was mediated by offspring BMI in early puberty and menarcheal age. Our observations align with the DOHaD hypothesis that intrauterine conditions shape long‐term reproductive health. Given the global rise in maternal overweight and obesity and their potential impact on the next generation's childhood growth and reproductive health, this is an area requiring further study. Future research should not only replicate and extend these findings in diverse populations but also explore potential biological mechanisms, including inflammatory pathways, that may underlie the observed associations. A deeper understanding of these processes is of importance for informing prevention and intervention strategies targeting early life origins of reproductive disorders such as dysmenorrhea.

## AUTHOR CONTRIBUTIONS

S. Zhou, C. Brouwer and T.G.M. Vrijkotte contributed to the conception of the study; S. Zhou and N. Shkarpa drafted the manuscript; T.G.M. Vrijkotte was responsible for data collection; S. Zhou and C. Brouwer performed the analysis; all the authors provided advice on the interpretation of the data; E. van den Boogaard, M. Finken and ThB. Twickler contributed to the constructive discussion; All authors contributed to the critical review and revise of the manuscript. All authors have stated for consents of publications.

## FUNDING INFORMATION

This work was financially supported by the Netherlands Organization for Health Research and Development (ZonMw) Grant (TOP, 40–00812–98‐11,010) and the China Scholarship Council (202206240018).

## CONFLICT OF INTEREST STATEMENT

The authors declare no conflict of interest related to this manuscript.

## Supporting information


Appendix S1.

## Data Availability

The data that support the findings of this study are available from the corresponding author upon reasonable request.
