# Supplementary material for: Clustered cardiometabolic risk in pregnancy and dysmenorrhea in offspring: Results from a prospective birth cohort study
Source: Eur J Clin Invest. 2025 Oct 14;56(1):e70134. doi: 10.1111/eci.70134 (PMC12817236; doi:10.1111/eci.70134)
Supplement: Supplementary file 1 — Appendix S1. [file ECI-56-e70134-s001.docx]

Table S1. An overview of all blood-derived and questionnaire-based variables in the Amsterdam Born Children and their Development study.

| Variable name | Source/  Instrument | Exact Wording of Question | Sample Size in the current study | Coding/Categories |
| --- | --- | --- | --- | --- |
| ***Exposure of interest*** |  |  |  |  |
| CCMR | Computed | / | 732 | CCMR is calculated by computing a z-score for the following components: Pre-pregnancy BMI, SBP, RGP, TG and ApoA1. |
| Pre-pregnancy BMI | Maternal questionnaire | 1. “How tall are you? (centimetres)”  2. “How was your weight the last time you checked this before the pregnancy? (kilogrammes)” | 982 | BMI is calculated by dividing a person's weight in kilograms by the square of their height in meters. This formula, using metric units, is: BMI = weight (kg) / [height (m)]². |
| DBP | Medical record | / | 869 | Continuous |
| SBP | Medical record | / | 869 | Continuous |
| RPG | Blood sample | / | 356 | Continuous |
| TG | Blood sample | / | 609 | Continuous |
| ApoA1 | Blood sample | / | 566 | Continuous |
| ***Outcome*** |  |  |  |  |
| Dysmenorrhea | Offspring questionnaire | “Do you suffer from menstrual pain (abdominal pain and/or back pain) during menstruation (period)?  Multiple options: no; yes abdominal pain; yes back pain; yes both.”  “Do you take medication for your period pain?  Multiple options: No I do not take any medication for my period; Yes I use painkillers such as paracetamol, Voltaren or Aleve Feminax during my period; Yes I was prescribed the contraceptive pills (the “Pill”) by my GP for my menstrual pain; Yes I use both for my menstrual problems.” | 982 | Categorical  The answer is recoded as no dysmenorrhea when answer is no pain and no medication taken;  The answer is recoded as dysmenorrhea when answer is with pain but no medication taken. |
| ***Covariates*** |  |  |  |  |
| Age at pregnancy | Maternal questionnaire | “How old are you? (years)” | 982 | Continuous |
| Gestational age at blood draw | Medical record | / | 982 | Continuous |
| Educational level | Maternal questionnaire | “How many years of education have you had after primary school? (years)  Or ‘option: I have only been to primary school.’” | 981 | Continuous |
| Ethnicity | Maternal questionnaire | “In which country were your own mother and father born?  Multiple options: The Netherlands; Surinam; The Antilles/Aruba; Turkey; Morocco; Ghana; other country: …” | 982 | Categorical  Western vs. Non-Western |
| Smoking during pregnancy | Maternal questionnaire | “Did you smoke cigarettes in the past week?  Multiple options: no; yes but less than one cigarette per day on average; yes on average … cigarettes per day.” | 982 | Categorical  The answer is recoded as no smoking when the answer is no;  The answer is recoded as smoking when the answer is yes but less than one cigarette per day on average or yes on average more than one cigarette per day. |
| Alcohol consumption during pregnancy | Maternal questionnaire | “Did you drink any alcohol in the past week?  Multiple options: no; yes but less than one glass per day on average; yes on average … glasses per day.” | 982 | Categorical  The answer is recoded as no drinking when the answer is no;  The answer is recoded as drinking when the answer is yes but less than one glass per day on average or yes on average more than one glass per day. |
| Anxiety level | STAI scale | The complete questionnaire([1](#_ENREF_1)) | 979 | Continuous |
| Depressive symptoms level | CES-D scale | The complete questionnaire([2](#_ENREF_2)) | 979 | Continuous |
| ***Mediators*** |  |  |  |  |
| BMI at early puberty | Multiple resources: national youth health care database and ABCD follow up physical check | / | 982 | BMI is calculated by dividing a person's weight in kilograms by the square of their height in meters. This formula, using metric units, is: BMI = weight (kg) / [height (m)]². |
| Age at menarche | Offspring questionnaire | 1. “Have you started your period yet?”  2. “How old were you when you first got your period? (years)” | 982 | Continuous |

SD: standardized deviation; IQR: interquartile range; STAI: the State-Trait Anxiety Inventory; CES-D: Centre for Epidemiologic Studies Depression scale; BMI: body mass index; DBP: diastolic blood pressure; SBP: systolic blood pressure; RPG: random plasma glucose; ApoA1: Apo lipoprotein A1; TG: triglycerides; CCMR: clustered cardiometabolic risk score.

Detailed and further information of the ABCD study have been published previously([3](#_ENREF_3)) and available at: <https://abcd.amsterdamumc.nl/> with an overview of data dictionary.

Table S2. Descriptive comparison of maternal cardiometabolic components in complete-case and 20 multiple-imputed datasets.

|  | Complete-case dataset | | Imputed datasets | |
| --- | --- | --- | --- | --- |
|  | N | mean±SD | N (each dataset) | mean±SD |
| Pre-Pregnancy BMI (kg/m^2^) | 982 | 22.8±3.5 | 982 | 22.8±3.5 |
| Blood pressure (mmHg)  -DBP  -SBP | 869 | 66.6±8.6  112.7±12.1 | 982 | 66.5±8.1  112.7±11.4 |
| RPG (mmol/L) | 356 | 4.4±1.0 | 982 | 4.4±1.0 |
| ApoA1 (gram/L) | 566 | 1.6±0.2 | 982 | 1.6±0.3 |
| TG (mmol/L) | 609 | 1.4±0.6 | 982 | 1.4±0.7 |

SD: standardized deviation; BMI: body mass index; DBP: diastolic blood pressure; SBP: systolic blood pressure; RPG: random plasma glucose; ApoA1: Apo lipoprotein A1; TG: triglycerides.

Significance level: ** p<0.05, *** p<0.01.

Table S3: Maternal clustered cardiometabolic risk score components in participants with and without dysmenorrhea (pooled results of 20 imputed datasets).

|  |  | Girls with dysmenorrhea (n=483) | Girls without dysmenorrhea (n=499) |
| --- | --- | --- | --- |
|  | Number of samples | mean±SD | mean±SD |
| Pre-Pregnancy BMI (kg/m^2^) | 982 | 23.1±3.7 | 22.6±3.2** |
| Blood pressure (mmHg)  -DBP  -SBP | 982 | 66.9±7.8  112.6±11.1 | 66.2±8.4  112.8±11.7 |
| RPG (mmol/L) | 982 | 4.4±1.0 | 4.4±1.0 |
| ApoA1 (gram/L) | 982 | 1.6±0.3 | 1.6±0.3 |
| TG (mmol/L) | 982 | 1.4±0.6 | 1.4±0.7 |
| CCMR (sum standardized z-scores) | 982 | 0.0±0.5 | 0.0±0.5 |

SD: standardized deviation; IQR: interquartile range; BMI: body mass index; DBP: diastolic blood pressure; SBP: systolic blood pressure; RPG: random plasma glucose; ApoA1: Apo lipoprotein A1; TG: triglycerides; CCMR: clustered cardiometabolic risk score. Difference between two groups was examined using independent t-test for continuous variables and Chi-square test for categorical variables.

Significance level: ** p<0.05, *** p<0.01.

Table S4. Multivariable logistic regression analysis of the association between individual and clustered maternal cardiometabolic risk z-scores (continuous) and the risk of dysmenorrhea in offspring aged 15 to 16 (pooled results of 20 imputed datasets).

|  | Model 1 | | | Model 2 | | |
| --- | --- | --- | --- | --- | --- | --- |
|  | OR | 95%CI | P value | OR | 95%CI | P value |
| CCMR (sum z-score) | 1.06 | 0.75-1.48 | 0.75 | 1.06 | 0.74-1.54 | 0.74 |
| Pre-pregnancy BMI (z-score) | 1.14 | 0.98-1.32 | 0.08 | 1.20 | 1.02-1.42 | 0.03** |
| DBP (z-score) | 1.13 | 0.95-1.34 | 0.15 | 1.09 | 0.92-1.30 | 0.32 |
| SBP (z-score) | 0.97 | 0.81-1.16 | 0.72 | 0.94 | 0.79-1.13 | 0.53 |
| RPG (z-score) | 1.03 | 0.80-1.33 | 0.82 | 1.02 | 0.78-1.34 | 0.90 |
| ApoA1 (z-score) | 0.96 | 0.76-1.22 | 0.70 | 0.97 | 0.75-1.26 | 0.79 |
| lnTG (z-score) | 0.99 | 0.80-1.23 | 0.94 | 0.96 | 0.78-1.19 | 0.72 |

OR: odds ratio; CI: confidence interval; Pre-pregnancy BMI: pre-pregnancy body mass index; SBP: systolic blood pressure; DBP: diastolic blood pressure; RPG: random plasma glucose; ApoA1: Apo lipoprotein A1; lnTG: natural log triglycerides; CCMR: clustered cardiometabolic risk.

Model 1: adjusted for gestational age of mothers at determinants measurements and age of girls at outcome measurement.

Model 2: adjusted for covariates in model 1 and maternal age at pregnancy, ethnicity, smoking/drinking behaviour, educational level, anxiety and depressive symptom scores.

Significance level: ** p<0.05, *** p<0.01.

References

1. Spielberger CD, Gonzalez-Reigosa F, Martinez-Urrutia A, Natalicio LF, Natalicio DS. The state-trait anxiety inventory. Revista Interamericana de Psicologia/Interamerican journal of psychology. 1971;5(3 & 4).

2. Lewinsohn PM, Seeley JR, Roberts RE, Allen NB. Center for Epidemiologic Studies Depression Scale (CES-D) as a screening instrument for depression among community-residing older adults. Psychology and aging. 1997;12(2):277.

3. van Eijsden M, Vrijkotte TG, Gemke RJ, van der Wal MF. Cohort profile: the Amsterdam Born Children and their Development (ABCD) study. International journal of epidemiology. 2011 Oct;40(5):1176-86. PubMed PMID: 20813863. Epub 2010/09/04. eng.
